# Mental health evaluation in senior year medical students

**DOI:** 10.1192/j.eurpsy.2023.1918

**Published:** 2023-07-19

**Authors:** M. A. Ghrab, I. Sellami, A. Haddar, M. Hajjaji, K. Jmal Hammami, M. L. Masmoudi

**Affiliations:** 1 Occupational medicine, University Hospital Hedi Chaker, Sfax, Tunisia; 2 University of Sfax, Sfax, Tunisia

## Abstract

**Introduction:**

Medical studies are known for being difficult and hard. They require a lot of dedication and hard work from students. This takes a toll on their mental health over the years.

**Objectives:**

Detect signs of anxiety, depression, and stress levels in 5^th^-year medical students.

**Methods:**

This study was cross-sectional through a self-administered pre-established questionnaire for medical students during September and October 2022. We used the General Anxiety Disorder-7 (GAD-7) questionnaire, the Patient Health Questionnaire-9 (PHQ-9) and the Perceived Stress Scale-10 (PSS-10). The satisfaction level with the choice of the medical field as a career was assessed on a scale ranging from 1 to 10.

**Results:**

Our population consisted of 54 5^th^-year medical students. The average age was 22.94±0.78 and 64.8% were female. All our population were singles. Active smokers represented 9.3% and alcohol consumption was reported by 9.3% of the participants. More than half of the population (51.9%) had a regular leisure activity. Most of the students had no medical history (72.2%) or psychiatric history (94.4%). The mean satisfaction level from choosing the medical field was 7.43±1.84. The mean GAD-7 and PHQ-9 scores were respectively 3.50±3.80 and 4.70±4.42. The PSS-10 score had a mean of 14.07±5.29. Five students (7.4%) presented self-harm thoughts. Students who were less satisfied with their choice of the medical field as a career had significantly higher scores of PHQ-9 (p<0.001), GAD-7 (p=0.004) and PSS-10 (p=.042).

**Conclusions:**

Medical studies are the first step for these young doctors in their professional careers, which presents further psychological stressors. More attention towards the mental health of this population is needed to properly prepare them for their future.

**Disclosure of Interest:**

None Declared

